# 768 The Significant Impact of Having a Dedicated Ambulatory Clinical Social Worker in the Burn Clinic

**DOI:** 10.1093/jbcr/irac012.321

**Published:** 2022-03-23

**Authors:** Margaret Spotts, Margaret M Dines, Crystal Foster, Arek J Wiktor, Elizabeth B Weber

**Affiliations:** UC Health Burn and Frostbite Center - Anschutz Medical Campus, Denver, Colorado; University Of Colorado Hospital, Aurora, Colorado; University of Colorado Hospital, Aurora, Colorado; University of Colorado Burn Center, Aurora, Colorado; UC Health Burn Center, Denver, Texas

## Abstract

**Introduction:**

A licensed clinical social worker (LCSW) has been recognized as an essential role within the Burn Center inpatient multidisciplinary team. However, for ambulatory patients facing acute crisis, resources can be scarce; relying on clinic nursing staff or an inpatient LCSW to problem solve psychosocial concerns can leave large gaps of patient care unaddressed. An Ambulatory LCSW (ALCSW) assists transition of care working to prevent unnecessary Emergency Room visits and readmissions. We sought to examine our experience utilizing an ALCSW in the Burn Center.

**Methods:**

A full-time burn ALCSW position was approved in fall of 2020 at our ABA verified Burn Center. The role includes extensive assessments of mental health, substance use, domestic violence, and safety concerns, connecting clinic patients to resources for transportation, benefits, insurance coverage, employment, food, and housing. The ALCSW also conducts short term patient centered, family & group therapy sessions. A retrospective review was performed on duties of the ALCSW comparing 9 months (December-August) pre and post hire. Data collected included visit types, hours of patient care, and interventions performed.

**Results:**

During the first 9 months, the ALCSW completed 1,008 patient encounters (approximately 25 hours of direct patient care per week) and 510 psychosocial assessments. In the first three months alone 258 substance use screens, 166 mental health assessments, 44 ASD/PTSD therapy sessions, 205 Homeless Shelter referrals and 128 community resource encounters were performed that would have otherwise been left unaddressed or added to the workload of our Burn Clinic RN or provider.

The ALCSW was able to see up to 89.39% more patients per month (n=89) compared to the inpatient LCSW who only responding to clinic patients with emergent needs (n=13). The increase of ALCSW encounters showed a 35.37% increase of completed visits without IP admission (n=pre 665 vs post 1029), 39.73% decrease of IP admissions after clinic visit (n=pre 73 vs post 44), and 17.82% increase of patients without inpatient admissions entirely (n=pre 572 vs post 696). In addition, the added effort of an ambulatory LCSW, has increased SOAR support group participation by 60.13% in calendar year 2021 compared to calendar year 2020 (n= pre 63 vs post 158).

**Conclusions:**

Hiring a dedicated Burn ALCSW can substantially increase the resources available to outpatients, fill voids of patient care, limit unnecessary hospital resources, and prevent admissions.

